# Health risk behavior among chronically ill adolescents: a systematic review of assessment tools

**DOI:** 10.1186/s13034-017-0172-5

**Published:** 2017-07-17

**Authors:** Derrick Ssewanyana, Moses Kachama Nyongesa, Anneloes van Baar, Charles R. Newton, Amina Abubakar

**Affiliations:** 1 Centre for Geographic Medicine Research Coast, Kenya Medical Research Institute, Kilifi, Kenya; 2 Utrecht Centre for Child and Adolescent Studies, Utrecht University, Utrecht, The Netherlands; 3 Department of Psychiatry, University of Oxford, Oxford, UK; 4 Department of Public Health, Pwani University, Kilifi, Kenya

**Keywords:** Health risk behavior, Adolescents, Chronic illness, Assessment tools, Lifestyle, Tool adaptation

## Abstract

**Background:**

Adolescents living with chronic illnesses engage in health risk behaviors (HRB) which pose challenges for optimizing care and management of their ill health. Frequent monitoring of HRB is recommended, however little is known about which are the most useful tools to detect HRB among chronically ill adolescents.

**Aims:**

This systematic review was conducted to address important knowledge gaps on the assessment of HRB among chronically ill adolescents. Its specific aims were to: identify HRB assessment tools, the geographical location of the studies, their means of administration, the psychometric properties of the tools and the commonest forms of HRB assessed among adolescents living with chronic illnesses globally.

**Methods:**

We searched in four bibliographic databases of PubMed, Embase, PsycINFO and Applied Social Sciences Index and Abstracts for empirical studies published until April 2017 on HRB among chronically ill adolescents aged 10–17 years.

**Results:**

This review indicates a major dearth of research on HRB among chronically ill adolescents especially in low income settings. The Youth Risk Behavior Surveillance System and Health Behavior in School-aged Children were the commonest HRB assessment tools. Only 21% of the eligible studies reported psychometric properties of the HRB tools or items. Internal consistency was good and varied from 0.73 to 0.98 whereas test–retest reliability varied from unacceptable (0.58) to good (0.85). Numerous methods of tool administration were also identified. Alcohol, tobacco and other drug use and physical inactivity are the commonest forms of HRB assessed.

**Conclusion:**

Evidence on the suitability of the majority of the HRB assessment tools has so far been documented in high income settings where most of them have been developed. The utility of such tools in low resource settings is often hampered by the cultural and contextual variations across regions. The psychometric qualities were good but only reported in a minority of studies from high income settings. This result points to the need for more resources and capacity building for tool adaptation and validation, so as to enhance research on HRB among chronically ill adolescents in low resource settings.

## Background

Research focusing on health risk behaviors (HRB) among adolescents living with chronic illness has increased over the past few decades. HRB are defined as specific forms of behavior associated with increased susceptibility to a specific disease or ill health on the basis of epidemiological or social data [1]. Examples of HRB include: alcohol, tobacco and drug use, unhealthy dietary habits, sexual behaviors contributing to unintended pregnancy and sexually transmitted diseases, behavior that contributes to unintentional injury or violence, and inadequate physical activity [2, 3]. In the past, it was presumed that chronically ill adolescents are restricted by their ill health from engaging in HRB [4, 5]. However, a growing body of evidence shows that chronically ill adolescents engage in such behavior at rates equivalent to [6–8] or at times higher [9–12] than their healthy peers. Some studies for example report higher frequency of cigarette smoking among adolescents with asthma [13, 14] and more substance or drug use among adolescents with mental illnesses [9, 15] compared to their healthy peers. In addition, chronically ill adolescents are often victims of behaviors resulting in unintentional injury and violence, such as bullying and sexual assault [16, 17]. Other problematic forms of HRB among chronically ill adolescents include; inadequate physical activity [18–20], risky sexual behavior [10, 11], and poor dietary habits [21].

Engagement in HRB is problematic for chronically ill adolescents because it hinders optimal care and management of ill health [22]. For example, studies among young people living with HIV report that anti-retroviral therapy adherence rates are poorer among the patients with riskier health lifestyle as compared to their HIV infected peers who have healthier lifestyles [23, 24]. Similarly, engagement in HRB such as tobacco use, recreational drugs use, and risky sexual behavior has been shown to hamper proper management of type 1 diabetes [25], asthma [26], and mental illness [27] among adolescents. Poor disease management compounded by direct adverse effects resulting from engagement in HRB, most likely translates into poorer health outcomes among chronically ill adolescents [5, 28]. Thus, promotion and maintenance of healthier behavioral practices early in adolescence has great potential to enhance positive long-term health outcomes for these patients [23].

Regarding the public health burden posed by HRB, frequent monitoring of such behaviors is recommended for supporting clinical and preventive efforts directed at improving lives of young people with chronic illnesses and their families [5, 29]. Although there are numerous measures of HRB, evidence is still meagre on the most frequently utilized HRB measures as well as the psychometric properties of HRB tools among chronically ill adolescents in various geographical contexts. Moreover, without proper adaptation, measurement bias and compromise to various psychometric properties like validity and reliability may arise [30, 31]. Bias also arises from unfamiliar content of the tests, translation challenges and unfamiliar means of tool administration [30]. Studies have similarly shown that variations in how questions are administered and how respondents are contacted affects the accuracy and quality of data collected [32]. There is still a lack of knowledge concerning the major forms of HRB, their commonly utilized assessment tools, their psychometric properties and their methods of administration in studies among chronically ill adolescents.

We therefore carried out this review to determine the current gaps in knowledge about tools to measure HRB. The review synthesizes findings from empirical studies conducted globally among adolescents living with chronic illnesses so as to: (i) identify the commonly utilized HRB assessment tools or sources of items used; (ii) describe the geographical utility of HRB assessments tools; (iii) identify the common means of HRB tool administration; (iv) document the reported adaptation and psychometric properties of HRB assessment tools or items; and (v) summarize the commonly assessed forms of HRB. We expect the results of this systematic review to aid HRB tool adaptation and validation procedures as well as enhance planning of research and interventions targeting adolescents living with chronic illnesses especially in low and middle income settings.

## Methods

This systematic review was conducted following recommended guidelines for conducting systematic reviews [33]. We searched for relevant literature in four bibliographic databases: PubMed, Embase, PsycINFO and Applied Social Sciences Index and Abstracts. The search was initially conducted between November and December 31, 2015 and later updated in May 2017. The search strategy was formulated by two reviewers (DS and AA) and comprised of the following non-MeSH terms combined with Boolean operators: *risk behavior* OR *risk taking* OR *health behavior* OR *healthy lifestyle* AND *adolescents* OR *Youth* OR *Teens* AND *Chronic condition* OR *Chronic disease* OR *Chronic illness*. Additionally, other relevant studies were identified by searching the reference lists of the retrieved articles.

In this review, our study inclusion criteria were: (i) empirical studies published in a peer reviewed journal from January 1, 1980 to April 30, 2017; (ii) studies with participants aged 10–17 years or with mean age within this age bracket; and (iii) studies assessing for both HRB and chronic illness among the same study participants. The chronic conditions considered are those documented by the United States Department of Health and Human Services for the standard classification scheme [34]. Only studies published in English were included in this review. Studies were excluded if: (i) they were non-empirical (such as reviews, commentaries, letters to editor, conference abstracts), (ii) their participants had an age range or mean age below or above the 10–17 years’ category and (iii) they assessed only HRB without consideration of chronic illness or vise-versa.

Data extraction was done by two independent reviewers (DS, MKN). The data was extracted to Microsoft Excel spread sheets with the following details from eligible studies: author and date of publication, country where the study was conducted, age of the participants (mean age), form of chronic illness, assessment tool or source of items on HRB, methods of administration of HRB measures, psychometric properties of the tool (if documented), and form of HRB assessed were extracted. For reliability, we extracted measures of internal consistency, and interrater reliability such as the Cronbach’s alpha, intra-class coefficient (ICC) and coefficient of correlation whenever reported. For tool validity, we extracted construct, criterion, divergent or convergent validities whenever reported. We also noted any aspects of tool adaptation such as cultural adaptation, content validity, forward-back translations in case they were reported (refer to Table 4).

Data analysis involved collating and summarizing of results. The synthesis of data extracted from the eligible studies was done narratively. Frequencies and/or percentages were computed in Microsoft Excel program so as to summarize the findings on: the frequency of the various HRB tools/measures reported in studies, geographical utilization of these tools, forms of HRB assessed, methods of HRB tool/item administration and the various chronic conditions reported. Due to the high variation in HRB tools or items used, the tools were classified into four categories namely: (i) full version HRB assessment tools; (ii) modified version of HRB assessment tools; (iii) borrowed items on HRB; and (iv) items on HRB either newly developed or whose source is not specified by the author. Also in situations where more than one eligible manuscript was written using data from the same study, frequencies on HRB tools were collated in order to represent a single frequency count for this reported HRB assessment tool. For purposes of data management the reported chronic conditions were re-categorized into: respiratory, cardio-vascular, metabolic, hematological, mental, musculoskeletal, neurologic, dermatologic, digestive, physical disability and HIV.

## Results

**Fig. 1:**
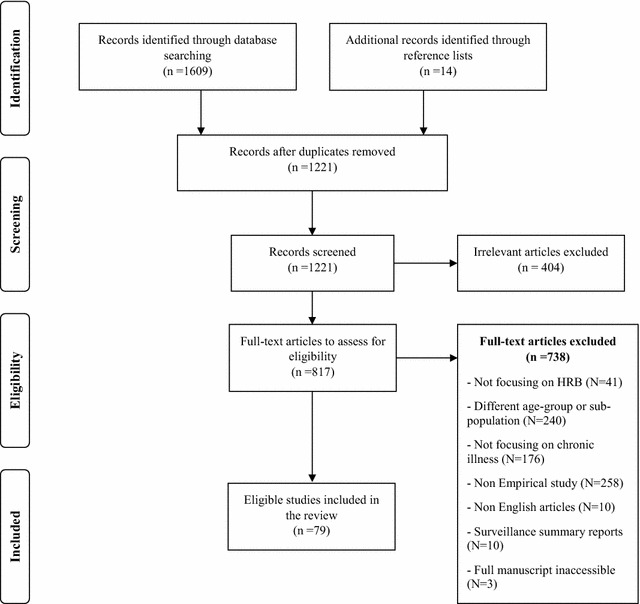
A flow diagram representing the article screening process of this review

The literature search yielded a total of 1623 articles and following a systematic appraisal of this literature (refer to Fig. 1), a total of 79 full articles were eligible for inclusion in this review.

Majority of the eligible studies were conducted in North America (60%) and Europe (24%). The rest of them were from Asia (8%), South America (2%), Oceania (2%) and a few were multi-site studies conducted in both Europe and North America (2%). The study site of one eligible study was not reported in the article [35].

Results of the most frequently utilized HRB tools/items are shown in Table 1. Briefly, from a total of 37 full version HRB tools, 7 tools namely: Health Behavior in School-aged Children (HBSC), Youth Risk Behavior Surveillance System (YRBSS), Korea Youth Risk Behavior Web-based Survey (KYRBS), Swiss Multi-centric Adolescent Survey on Health (SMASH), car, relax, alone, forget, friends, trouble (CRAFT) substance Abuse Screening Test, Alcohol Use Disorder Identification Test (AUDIT) and Life and Health in Youth questionnaire were the most commonly utilized. The items on HRB in 12 of the studies from this review were either newly developed or their sources were not specified [23, 36–46].

**Table 1:** Frequency of utilization of HRB tools and sources of items

HRB tools or items	Frequency (%)
(i) Full version of HRB tool (n = 37)	
Health Behavior in School-aged Children (HBSC)	4 (8.2)
Youth Risk Behavior Surveillance System (YRBSS)	3 (6.1)
Korea Youth Risk Behavior Web-based Survey	3 (6.1)
CRAFT substance Abuse Screening Test	3 (6.1)
Swiss Multi-Centre Adolescent Survey on Health (SMASH) questionnaire	2 (4.1)
Alcohol Use Disorder Identification Test (AUDIT)	2 (4.1)
Life and Health in Youth questionnaire	2 (4.1)
Other tools (n = 30)	30 (61.2)
(ii) Source of borrowed HRB items (n = 14)	
Youth Risk Behavior Surveillance System (YRBSS)	8 (29.6)
Health Behavior in School-aged Children (HBSC)	4 (14.8)
Child Behavior Checklist	3 (11.1)
Youth Self Report	2 (7.4)
Other sources (n = 10)	10 (37.1)
(iii) Modified version of HRB assessment tools (n = 3)	
Modified Youth Risk Behavior Surveillance System	1 (33.3)
Modified Self Report of Delinquency	1 (33.3)
Modified Michigan Alcohol Screening Test (MAST)	1 (33.3)
(iv) Items newly developed or with unspecified source (n = 12)	12 (100)

The HBSC tool is a self-completion questionnaire administered in class room settings to adolescents aged 11–15 years and the HBSC study is conducted every 4 years across 44 countries in Europe and North America since its inception in 1982 [3]. The key health behaviors captured by this tool include; bullying and fighting, oral hygiene, physical activity and sedentary behavior, sexual behavior, substance use (e.g. alcohol, tobacco and cannabis), weight reduction behavior, behaviors resulting in injury, and dietary habits [3]. The YRBS tool (Standard and National High School questionnaires) is developed by the US Centers for Disease Control and Prevention (CDC) to monitor HRB that are considered leading causes of disability, death and social problems among youths in 9th to 12th grade (approximately 14–18 years) in the US Students complete the self-administered questionnaire during one class period and record their responses directly in an answer sheet. This tool assesses 6 forms of HRB: sexual risk behaviors, tobacco use, alcohol and other drug use, inadequate physical activity and unhealthy dietary behaviors [2].

Results on the most frequently assessed forms of HRB are summarized in Table 2. Overall, alcohol, tobacco and other drug use and physical inactivity were the most frequently assessed forms of HRB.

**Table 2:** Frequency of HRB assessed among chronically ill adolescents

Forms of HRB assessed	Frequency (%)
Smoking	49 (18.9)
Alcohol use	42 (16.2)
Physical inactivity	35 (13.5)
Drug and other substance use	34 (13.1)
Sexual risk behavior	20 (7.7)
Violence/aggressive/anti-social behavior	26 (10.0)
Poor dietary behavior	18 (6.9)
Self-harm	12 (4.6)
Sedentary behavior	9 (3.5)
Behavior resulting to unintentional injuries	5 (1.9)
Inadequate sleep behavior	6 (2.3)
Poor hygiene	2 (0.8)
Sun exposure behavior	1 (0.4)

The HRB tool/item administration (Table 3), adolescent self-completed paper and pencil format, face-to-face interview with the adolescent, and Audio Computer Assisted Self Interview (ACASI) were the most frequently utilized means.

**Table 3:** A summary of methods for administration of HRB tools or items

Method of HRB tool/item administration	Frequency (%)
Adolescent self-completed paper and pencil format	41 (49.4)
Face-to-face interview with the adolescent	10 (12.0)
Audio Computer Assisted Self Interview (ACASI) or Computer Assisted Personal Interview (CAPI)	7 (8.4)
Online questionnaire	5 (6.0)
Telephone administered to the adolescent	5 (6.0)
Mailed questionnaire	4 (4.8)
Face-to-face interview with adolescent and parent/guardian	3 (3.6)
Face-to-face interview with parent/guardian	2 (2.4)
Parental filled questionnaire	2 (2.4)
Telephone delivered to parent/guardian	1 (1.2)
Means not specified	3 (3.6)

Adaptation or psychometric properties of the HRB tools or items among the study population were only reported in 17 studies moreover. Most of these (82%) were conducted in the USA (see Table 4). Five of these studies reported aspects of adaptation such as forward-back translations, content validity, item completeness, and cultural appropriateness but without reporting any psychometric data [44, 47–50]. Among those that reported psychometric data, only 6 studies [9, 18, 51–54] reported this data for an entire HRB tool or entire tool from which HRB items were borrowed while the rest reported only data for select items from the HRB tool. Psychometric data for the whole HRB tool was reported for the following instruments: Kriska’s Modifiable Activity questionnaire; Modified Self Report of Delinquency; Risk Behavior and Risk Scale; Delinquency Scale; and the Denys Self-Care Practice instrument. Moreover, psychometric properties of Youth Self Report; Child Behavior Check List; and the Structured Clinical Interview for the DSM-IV in the context of HRB evaluation were also reported. The reported psychometric properties of these tools satisfied the recommended thresholds for psychometric rigor for example the internal consistency (coefficients ranged from 0.73 to 0.98) and test–retest reliability (coefficients ranged from 0.58 to 0.85). The psychometric data reported on selected HRB items were mainly for items assessing physical activity or sedentary behavior [38, 55] and these also had good test–retest reliability ranging from 0.8 to 0.81 and good internal consistency of 0.73.

**Table 4:** A summary of data extracted from the eligible studies included in this review

Author	Country	Age/mean age (years)	illness	Form of HRB	HRB tool or source of HRB items	Adaptation and psychometric properties
Holmberg and Hjern [71]	Sweden	10	ADHD	Behavior resulting into violence	Items adapted from HBSC	NR
Husarova et al. [72]	Slovakia	13–15	Asthma, learning disability or presence of a long term illness	Sedentary lifestyle	Health Behaviour in School-aged Children	NR
Park et al. [21]	USA	15–17	Asthma	Tobacco smoking, poor dietary habits	2009 YRBSS questionnaire	Convergent validity: the item on soda-intake from the questionnaire correlated with soda intake from 24 h dietary recalls (r = 0.44)
Kim et al. [73]	Korea	13–18	Asthma	Tobacco use, physical inactivity, sedentary life-style	2007 Korea Youth Risk Behavior Web-Based Survey (KYRBWS)	NR
Rhee et al. [13]	USA	16	Asthma	Tobacco use, illicit substance/drug use, alcohol drinking	Periodic Assessment of Drug Use (PADU)	NR
Jones et al. [8]	USA	14–18	Asthma	Physical inactivity, sedentary lifestyle	2003 YRBSS	NR
Jones et al. [14]	USA	14–18	Asthma	Tobacco smoking, drug/substance use	2003 YRBSS	NR
Tercyak [74]	USA	16.1	Asthma	Tobacco smoking behavior	Adapted from the YRBSS	NR
Swahn and Bossarte [16]	USA	14–18	Asthma	Behavior resulting into violence	2003 YRBSS	NR
Lee and Shin [75]	Korea	12–17	Atopic dermatitis depression	Self-harm, poor sleep behavior, behavior resulting to violence, alcohol drinking, tobacco smoking, physical inactivity	Korean Youth Risk Behavior Survey 2013 (KYRBS)	NR
Oh et al. [76]	Korea	14.8	Atopic disease (asthma, allergic rhinitis, atopic dermatitis)	Poor sleep behavior	Korean Youth Risk Behavior Survey 2013 (KYRBS)	NR
Lunt et al. [60]	Australia	14.6	Cardiac disease	Physical inactivity	Items adapted from New South Wales Schools Fitness and physical activity survey	NR
Barbiero et al. [19]	Brazil	2–18	Congenital heart disease	Tobacco smoking, physical inactivity	International Physical Activity Questionnaire (IPAQ)	NR
Uzark et al. [42]	USA	16.1	Congenital heart disease	Sexual risk behavior, tobacco smoking, alcohol drinking, physical inactivity	Source of items not clear	NR
Nixon et al. [18]	USA	7–17	Cystic fibrosis	Physical inactivity	Kriska’s Modifiable Activity questionnaire	Convergent validity: physical activity measured by HRB tool correlated significantly with measurements by a Caltrac motion sensor (r = 0.4, p = 0.04) Test–retest reliability: a 3 months period test–retest reliability was ICC = 0.77, 0.70, 0.58 for 3 levels of physical activity
Adrian et al. [77]	USA	15–19	Depression	Tobacco smoking, drug/substance use, alcohol drinking, poor dietary, physical inactivity, poor sleep behavior	Washington State Healthy Youth Survey	NR
Lampard et al. [78]	USA	14.4	Depression	Poor dietary habits, tobacco smoking	EAT 2010 Survey Tool	EAT 2010 Survey Tool was first pilot tested with 129 students Test–retest reliability of the item used to capture any of these behaviors was ICC = 0.85
Frazer et al. [54]	USA	16.1	Depression	Anti-social acts (delinquent behavior), drug/substance use, alcohol use	Delinquency scale	Internal consistency (Cronbach’s alpha = 0. 84)
Allison et al. [79]	Canada	12–17	Depression	Physical inactivity	Items extracted from the YRBSS	NR
Tortolero et al. [37]	USA	11.2	Depression	Behavior resulting into violence	Source of items is not clear	NR
Dube et al. [80]	USA	12–17	Depression	Tobacco smoking	National Health and Nutrition Examination Survey	NR
Richardson et al. [81]	USA	13–17	Depression	Drug/substance use, alcohol drinking	CRAFT substance Abuse Screening Test	NR
Katon et al. [15]	USA	13–17	Depression	Tobacco smoking, drug/substance use, alcohol drinking, poor dietary habits, physical inactivity, sedentary lifestyle	CRAFT substance Abuse Screening Test	NR
Simpson et al. [12]	Canada	14–18	Depression	Sexual risk behavior, tobacco smoking, drug/substance use, alcohol drinking, poor dietary habits, physical inactivity, unintentional injuries, behavior resulting into violence	2001/2 HBSC	Construct validity: a one-factor solution with loadings 0.63–0.80 indicated the following items: lifetime cannabis use; unprotected sexual intercourse; lifetime use of other illicit drugs; lifetime drunkenness; and present smoking status. Internal consistency: an excellent Cronbach’s alpha = 0.81 was obtained for the entire HRB tool
Tercyak et al. [82]	USA	14.1	Depression	Tobacco smoking, physical inactivity, sun protective behavior	Items derived from Youth Risk Behavior Survey (YRBSS)	NR
Elder et al. [55]	USA	15.5	Depression	Tobacco use, alcohol drinking, poor dietary habits, physical inactivity, sedentary lifestyle	Items adapted from: 1997 YRBSS, 24 h food intake record (FIR), 7 day physical activity recall	Inter-observer reliability for FIR was r = 0.72 for 12 key nutrients Test–retest reliability of the items on TV watching in terms of total hours per week was 0.80 at pilot testing
Pronk et al. [83]	USA	13–17	Depression	Tobacco smoking, alcohol drinking, poor dietary habits, physical inactivity,	Items adapted from: Behavior Risk Factor Surveillance System and from Recommended Food Score	NR
Brooks et al. [47]	USA	14–18	Depression	Sexual risk behavior, tobacco smoking, alcohol drinking, substance/drug use, poor dietary, physical inactivity, behavior resulting into violence	Massachusetts Adolescent Health Survey	The tool was reviewed by academic experts, adolescent health practitioners and survey researchers for content validity and cultural appropriateness HRB items were pilot-tested among 4 adolescent focus groups and were pre-tested for clarity, length and completeness of closed ended questions
Schmitz et al. [38]	USA	11–15	Depression	Physical inactivity, sedentary lifestyle	Source of items not clear	The test–retest reliability for the item on physical activity was 0.65 The test–retest reliability for sedentary lifestyle was 0.81 and a Cronbach’s alpha of 0.73
Shrier et al. [39]	USA	17.1	Depression	Sexual risk behavior, drug/substance use, alcohol drinking	Source of items not clear	NR
Moradi-Lakeh et al. [84]	Saudi Arabia	15–19 (majority)	Diabetes mellitus, congestive heart failure, renal failure, cancer	Physical inactivity, sedentary lifestyle, poor dietary habits, tobacco smoking, unintentional injuries	Saudi Health Information Survey (SHIS)	NR
Ohmann et al. [85]	Austria	9–19	Diabetes	Anti-social acts	Child Behavior Checklist, Youth Self Report	NR
Scaramuzza et al. [6]	Italy	14	Diabetes	Sexual risk behavior, self-harm, tobacco smoking, alcohol drinking, substance/drug use	Items adapted from YRBSS	NR
Kyngas [36]	Finland	13–17	Diabetes	Tobacco smoking, alcohol drinking, physical inactivity	A newly developed questionnaire	NR
Soutor et al. [86]	USA	9–17	Diabetes	Poor dietary habits, physical inactivity	24 h recall interviews	NR
Gold and Gladstein [35]	Not stated	15	Diabetes	Tobacco smoking, substance/drug use, alcohol drinking	Modified Michigan Alcohol Screening Test	NR
Timko et al. [87]	USA	10–11	Juvenile rheumatic disease	Tobacco smoking, drug/substance use, alcohol drinking,	Health and daily living form	NR
MacDonell et al. [88]	USA	15.8	HIV	Substance use	The car, relax, alone, forget, friends, trouble (CRAFT)	NR
Elkington et al. [89]	USA	9–16	HIV	Sexual risk behavior, tobacco smoking, alcohol drinking, substance/drug use	Adolescent Sexual Behavior Assessment (ASBA), Diagnostic Interview Schedule for Children-IV	NR
Lagrange et al. [23]	USA	17.2	HIV	Poor dietary habits, physical inactivity, poor sleep behavior	Six questions with unclear sources	NR
Asnani et al. [48]	Jamaica	17	Sickle cell disease	Sexual risk behavior, tobacco smoking, drug/substance use, alcohol drinking	Jamaican Youth Risk and Resilience Behavior Survey	Validity of instrument was assured through pretesting it among a youth group and a panel of adolescent health experts
AlBuhairan et al. [49]	Saudi Arabia	15	Mental illness, asthma, hematological disorders, skin disorders, genito-urinal disorders	Tobacco smoking, drug/substance use, alcohol drinking, poor dietary habits, physical inactivity, sedentary lifestyle, unintentional injuries, behavior resulting into violence	Items adapted from YRBSS and Global School-based Student Health Survey	Items underwent cultural adaptation and culturally inappropriate items were excluded (e.g. on sexual behavior and sexually transmitted infections)
Kline-Simon et al. [46]	USA	15	Mental illness conditions (depression, bipolar spectrum disorders, personality disorders, dementia, schizophrenia, other psychoses) Asthma, sinusitis, arthritis, rhinitis, diabetes mellitus, inflammatory bowel disease, migraine	Substance use	Source of items not clear	NR
Kunz et al. [43]	USA	16.1	Cystic fibrosis, inflammatory bowel disease, arthritis, hematologic condition, cardiac condition	Tobacco smoking, alcohol drinking	Source of items not clear	NR
Conner et al. [90]	USA	15.9	HIV, Depression	Tobacco smoking, alcohol drinking, substance/drug use	Items adapted from Reaching for Excellence in Adolescent Care and Health (REACH)	NR
Olsson et al. [91]	Sweden	15–16	Rheumatism, autism, epilepsy, diabetes, ADHD, eczema, mental problem, asthma, visual/speech impairment, dyslexia	Poor dietary habits, physical inactivity, behavior resulting into violence	2008 Ung I Värmland questionnaire	NR
Singh et al. [92]	USA	10–17	Asthma, autism, depression, ADHD, learning disability, hearing problems	Tobacco smoking, physical inactivity, sedentary lifestyle, poor sleep behavior	National Survey of Children’s Health questionnaire	NR
Woods et al. [51]	USA	11–16	Asthma, persistent bowel problems, diabetes, sickle cell anaemia, and others	Behavior resulting into violence	Youth Self Report (YSR), Child Behavior Checklist (CBCL), Modified Self Report of Delinquency (MSRD)	Test–retest reliability of YSR was r = 0.8 and internal consistence, Cronbach’s alpha = 0.96 Internal consistency of MSRD was Cronbach’s alpha = 0.98 The internal consistency of CBCL was Cronbach’s alpha = 0.91 and 0.80 for externalizing and internalizing sub-scales respectively
Wilens et al. [9]	USA	6–17	ADHD, depression	Tobacco smoking, alcohol drinking, drug/substance use	Structured Clinical Interview for the DSM-IV	Inter-rater reliability of the diagnosis procedures was assessed by comparing findings by assessment staff and those by certified child and adult psychologists who used the audio taped assessment interviews. Kappa coefficient for substance use disorder = 1.0
Bush et al. [41]	USA	11–17	Asthma, depression	Tobacco smoking	Source not clear	NR
Silburn et al. [93]	Australia	12–17	Asthma, visual and hearing impairment, learning difficulties, speech problems	Sexual risk behavior, tobacco smoking, drug/substance use, alcohol drinking, physical inactivity, self-harm	Western Australia Aboriginal Child Health Survey	NR
Suris and Parera [11]	Spain	16.1	Diabetes, asthma, epilepsy, scoliosis, cancer, arthritis	Sexual risk behavior, tobacco smoking, drug/substance use, alcohol drinking	Catalonia Adolescent Health Survey 2001	NR
Blum et al. [40]	USA	16.2	Physical disability, learning disability, emotional disability	Sexual risk behavior, tobacco smoking, alcohol drinking, self-harm, behavior resulting into violence	Source of items not clear	NR
Britto et al. [59]	USA	15.6	Cystic fibrosis, sickle cell disease	Sexual risk behavior, tobacco smoking, drug/substance use, alcohol drinking, unintentional injuries, behavior resulting into violence, self-harm	Modified version of YBS	NR
Choquet et al. [10]	France	16.2	Cancer, hemophilia, arthritis, nephropathy, diabetes, mental disease, metabolic disease, eczema, psoriasis, asthma, cardio-pathy	Sexual risk behavior	Items derived from HBSC and Choquet-Ledoux study	NR
Frey et al. [53]	USA	14.2	Diabetes, asthma	Sexual risk behavior, tobacco smoking, alcohol drinking, substance/drug use	Risky Behavior and Risk Scale	Internal consistency ranged from 0.85 to 0.95 for the three subscales of the HRB tool
Frey [52]	USA	9–16	Diabetes, asthma	Poor dietary habits, physical inactivity, poor sleep behavior	Denyes self care practice instrument	Internal consistency ranged from 0.73 to 0.79
Suris et al. [7]	USA	14–15	Scoliosis, arthritis, muscular dystrophy, diabetes, seizures, asthma	Sexual risk behavior	Minnesota Adolescent Health Survey 1986–7	NR
Nylanderet al. [61]	Sweden	15–18	Presence of at least one chronic disease	Sexual risk behavior, tobacco smoking, drug/substance use, alcohol drinking, physical inactivity, behavior resulting into violence, self-harm	2011 Life and Health in Youth questionnaire	NR
Warren et al. [44]	USA	16.6	Presence of comorbid chronic conditions	Poor dietary habits, behavior resulting into violence, poor hygiene practices	Items borrowed from previous population level surveys	Clarity and understandability of items assessed by expert panel review and cognitive interviews of adolescents
Ardic and Esin [94]	Turkey	16.0	Presence of any pre-existing or current chronic illness	Poor dietary habits, physical inactivity	Adolescent Lifestyle Profile Scale	NR
Nylanderet al. [95]	Sweden	15–18	Physical impairment or presence of a chronic disease (yes/no)	Sexual risk behavior, tobacco smoking, drug/substance use, alcohol drinking, behavior resulting into violence, self-harm, anti-social acts	2008 Life and Health in Youth questionnaire	NR
Santos et al. [96]	Portugal	15	Presence of a chronic disease (yes/no)	Alcohol use, behavior resulting into violence, self-harm	2010 Health Behavior in School-aged Children (HBSC)	NR
Sentenac et al. [50]	Multi-site (Europe and North America)	11–16	Presence of a chronic disease (yes/no)	Behavior resulting into violence	2005/6 HBSC	Language equivalence was ensured by translation and back translation
Rintala et al. [97]	Canada and Finland	13–15	Physical disability or presence of a chronic disease (yes/no)	Physical inactivity	Items adapted from 2001/2 HBSC Moderate-to-vigorous intensity physical activity screening measure	NR
Wilcox et al. [45]	USA	10.4	Physical disability or presence of a chronic disease (yes/no)	Self-harm, anti-social acts, sexual risk behavior, alcohol/substance use behavior	Source of items not clear	NR
Alriksson-Schmidt et al. [17]	USA	15–18	Presence of a chronic disease (yes/no)	Tobacco smoking, drug/substance use, alcohol drinking, behavior resulting into violence	2005 YRBSS	NR
Han et al. [98]	Korea	12–19	Presence of a chronic disease (yes/no)	Tobacco smoking, alcohol drinking, self-harm	2006 Korea Youth Behavioral Risk Factor Surveillance	NR
Jones and Lollar [20]	USA	14–18	Presence of a chronic disease (yes/no)	Sexual risk behavior, tobacco smoking, drug/substance use, alcohol drinking, poor dietary habits, behavior resulting into violence, self-harm	2005 YRBSS	NR
Suris et al. [29]	Switzerland	17.9	Presence of a chronic disease (yes/no)	Sexual risk behavior, tobacco smoking, drug/substance use, alcohol drinking, poor dietary habits, behavior resulting into violence, anti-social behavior	SMASH questionnaire	NR
Erickson et al. [67]	USA	14.9	Depression, presence of a chronic disease (yes/no)	Tobacco smoking, drug/substance use, alcohol drinking, self-harm	Items adapted from the Minnesota Student Survey	The internal consistency of the items on substance use behavior was Cronbach’s alpha = 0.79
Heflinger and Saunders [99]	USA	4–17	Depression, presence of a chronic disease (yes/no)	Anti-social acts	Child Behavior Checklist, Columbia Impairment Scale	NR
Haarasilta et al. [100]	Finland	15–19	Presence of at least one chronic illness, depression	Tobacco smoking, alcohol drinking, physical inactivity	1996 Finnish Health Care Survey questionnaire	NR
Mattila et al. [101]	Finland	12–18	Presence of a chronic disease (yes/no)	Tobacco smoking, drug/substance use, alcohol drinking, poor dietary habits, physical inactivity, behavior resulting into violence, poor hygiene/sanitation	1999 Adolescent Health and Life-style Survey questionnaire	NR
Huurre et al. [102]	Finland	16	Presence of at least one chronic illness, depression	Tobacco smoking, alcohol drinking, physical inactivity,	Alcohol Use Disorder Identification Test (AUDIT)	NR
Miauton et al. [103]	Switzerland	15–17 and 18–20	Presence of a chronic disease (yes/no)	Sexual risk behavior, tobacco smoking, drug/substance use, alcohol drinking, unintentional injuries, behavior resulting into violence	Swiss Multi-centre Adolescent Survey on Health (SMASH)	NR
Tremblay et al. [104]	Canada	12–17	Presence of at least one chronic illness, depression	Tobacco smoking, alcohol drinking, physical activity, poor dietary habits	Canadian Community Health Survey	NR
Huurre and Aro [105]	Finland	16	Presence of at least one chronic illness, depression	Tobacco smoking, alcohol drinking, physical activity	AUDIT	NR
Williams and Shams [106]	England	14–15	Presence of at least one chronic disease	Tobacco smoking, drug/substance use, alcohol drinking, physical inactivity	Health and Lifestyle Survey, London	NR

The HRB tools were largely used among adolescents with the chronic conditions of mental illness, especially depression (21.4%), respiratory conditions such as asthma and cystic fibrosis (13.8%), metabolic conditions such as diabetes (9.4%) and neurological conditions such as autism spectrum disorders, epilepsy and cerebral palsy (6.9%). To a lesser extent, the HRB tools were also utilized among adolescent patients with musculoskeletal conditions such as arthritis, cardio vascular conditions (e.g. congenital heart disease and hypertension), HIV, cancer, digestive tract conditions (e.g. inflammatory bowel disease and gastritis), disabling conditions (e.g. visual, speech and hearing problems) and dermatological conditions such as atopic dermatitis and eczema. The detailed summary of eligible studies is presented in Table 4.

## Discussion

This review identified the commonly utilized HRB assessment tools or sources of items used; describing the geographical utility of HRB assessments tools, the common methods of HRB tool administration, the adaptation and psychometric properties; and providing a summary of the forms of HRB commonly assessed. Our findings show that the YRBS and HBSC are the most frequently used tools to assess HRB or sources of items on HRB. This may partly be explained by their high level of comprehensiveness in assessing priority and multiple forms of HRB thereby being useful in many contexts. While both tools assess for HRB among adolescents, the YRBSS targets an older adolescent age group compared to the HBSC. The HBSC however focuses more on the social and environmental context for HRB such as influence of peers, school environment, and family characteristics. The YRBSS explores HRB in greater detail compared to the HBSC although the former lacks items on oral hygiene, health complaints and chronic illnesses. Besides the YRBSS and HBSC, a wide range of other HRB tools have been utilized, and some of them assess the same form of HRB but in a different format. One challenge that this may present is the lack of uniformity or standardized formats to compare similar HRB outcomes across different study populations.

Findings from this review also indicate that research on HRB among adolescents living with chronic illnesses in low and middle income countries (LMIC) is still limited. This is unfortunate since the majority of the adolescent population lives in LMICs [56] where a disproportionately higher burden of HRB occurrence is also reported [57]. There are three potential reasons that may explain the limited research on HRB among chronically ill adolescents in LMICs. First there is limited research that explicitly focuses on the adolescent age-group [5]. Second, research on this topic is not adequately prioritized [4]. Nonetheless, research on HRB among chronically ill adolescents has significantly grown over the past two decades [4, 5] though with disproportionately lower prioritization especially in LMICs. The third reason is the scarcity of standardized measures on various health outcomes among chronically ill adolescents [5]. The need for more investment in research on health and behavioral outcomes among chronically ill adolescents especially in LMICs cannot be overemphasized given that the burden of chronic diseases is increasing in such settings [58].

The use of appropriate and psychometrically sound instruments is essential for having good insight in adolescents’ behavior so as to be able to address certain forms of behavior that could be dangerous either for the patients themselves or for others. However, our findings indicate that HRB tool adaptation and psychometric properties are rarely reported among studies on HRB of chronically ill adolescents. Partly, this could be due to the fact that the majority of the studies were conducted in the western context where the majority of these tools have been developed. To indicate the adaptation and psychometric properties, some of the authors simply cited studies where similar HRB tools or items have been previously utilized [59–61]. This may not guarantee validity and reliability for a number of reasons. First, some of the tools were previously adapted and validated for use among adolescents without chronic conditions and thus we cannot ascertain if they retain their good psychometric properties when used among chronically ill adolescents. Secondly, some of the original validation or adaptation may have taken place more than two decades back and considering the evolution of HRB, various behavioral constructs used in these tools may no longer be appropriate. Another observation is that many researchers borrow specific items from previously well validated or standardized HRB tools but without checking the item specific psychometric properties. Our findings also reveal that there is a tendency for researchers to perform the adaptation processes such as forward-back translation and content review for item completeness, clarity or cultural appropriateness; without performing psychometric evaluations. It should be emphasized that much as adaptation is an important process, psychometric evaluation is equally critical for ascertaining item reliability and validity. Without adequate adaptation and psychometric evaluation we cannot ascertain if the scales and items retain their good psychometric properties following the modifications made. Overcoming such challenges requires a mixed methods approach for tool adaptation and validation [31, 62, 63]. For instance, a four step approach has been suggested as adequate for adapting tools in low and middle income countries [64]. The four step approach suggested for LMICs entails: (i) construct definition which can be done through review of literature, and consultation with community or local professionals in order to achieve conceptual clarity and equivalence; (ii) item pool creation which involves preparation of a list of potentially acceptable items in a clear and unambiguous language using feedback from the first step; (iii) developing clear guidelines for administration of the items to ensure operational equivalence; (iv) test evaluation which involves psychometric evaluation to assess measurement and functional equivalence [64].

Additionally, findings from this review indicate that there are numerous methods of HRB tool or item administration. Self-administered paper and pencil format was the most popular method and this could have been because of the participants’ good level of literacy given that majority of them were school attending adolescents. This method of administration is also preferred as it is associated with a high level of privacy and ease of administration [32]. On the contrary, its disadvantage arises from its requirement for some literacy levels among the respondents as well as the cognitive burden that respondents face in comprehending and recalling their experiences [32, 65]. Face-to-face interviews were also frequently utilized in assessing HRB. This method is linked to high response rates and the benefit of probing participants and clarifying unclear questions [65]. Nonetheless, face-to-face interviews are hampered by the lack of anonymity which may result to social desirability bias and impression management [32, 65]. Similar to findings from other studies [32, 66], our review shows that there is growing utilization of electronic methods of HRB tool and item administration. Electronic methods [such as the Audio Computer Assisted Self Interview (ACASI), telephone and internet based surveys] are valued for their high level of privacy or anonymity [32, 65] and some of them such as the ACASI have been further designed to benefit people with low literacy levels [65]. However, electronic methods require access to electronic devices and services (such as telephone, computer, and internet), may require greater auditory demands and some demand a high level of literacy [32, 66]. The presence of numerous HRB tool administration methods presents a wide set of options which can be tailored to suit contextual factors, research skills, resource availability and specific needs of study populations. However, researchers should carefully think through the dynamics surrounding tool administration and data collection procedures in order to identify the most appropriate methods to ensure that high quality data is collected.

Furthermore, our findings show that alcohol, tobacco, drug use behavior and physical inactivity are the most frequently researched HRB among adolescents with chronic conditions. Substance use among chronically ill adolescents is of major concern and many studies report higher or equivalent rates of substance use (e.g. cannabis, tobacco, illicit drugs) among these adolescents in comparison to their healthy peers [12, 13, 67]. This may explain why most of HRB research among this group focuses on substance use behavior. Our findings also indicate that physical inactivity and sexual risk behavior are frequently assessed. Growing research interest on sexuality of chronically ill adolescents indicates that sexual risk behavior is a concern [7, 10–12] and this dissents the earlier notion that they are less sexually active than their healthy peers [4]. Likewise, physical activity among adolescents with chronic conditions is gaining measureable research interest [28]. This may surround its vital role in appropriate management of chronic illness such as: cardio-respiratory fitness among asthmatic patients and optimization of quality of life among patients with cerebral palsy [28]. Our results also indicate that violence related behaviors are frequently investigated among chronically ill adolescents. Adolescents with chronic illnesses often fall victim of violence such as bullying, assault and forced sexual encounters [17, 50]; and thus raising the need for increased research on this matter. On the other hand, our findings show that poor hygiene, inadequate sleep and behavior resulting to unintentional injury were the least frequently assessed forms of HRB in this review. This may be due to the reality that most of these problematic behaviors are of greater research interest in LMICs (whose representation is still low) where their occurrence is documented to be greater, compared to high income settings [57, 68]. Our findings on the variation in the frequency of the forms of HRB assessed, may partly imply that there is some tendency to measure HRB in isolation. However, co-occurrence of different adolescent HRB is increasingly documented [69, 70], and therefore different forms of HRB should be assessed concurrently.

Our review draws its major strengths from the utilization of a rigorous methodological framework [33] and also its specific focus on the adolescent age-group in a global perspective. However, we did not appraise the quality of the studies included in our systematic review. Nonetheless, given that our study objectives aimed at describing extent of utilization of HRB tools and providing an over-view of various forms of HRB assessed, we do not expect any major issues arising from the quality of studies to influence our findings.

## Conclusion

Overall, most research on health risk behavior among chronically ill adolescents emanates from high income settings such as Europe and North America where the majority of the HRB assessment tools have also been developed. Therefore more investment is needed in research on health and behavioral outcomes among chronically ill adolescents especially in LMICs. Although the YRBSS and HBSC are utilized most, a variety of other HRB tools are used as well, however without documentation of adaptation and psychometric qualities. This poses challenges for researchers and practitioners who are keen to evaluate HRB in LMICs. We recommend the use of the mixed methods approach for tool adaptation and validation, which involves both qualitative approaches (e.g. focus group discussions and in-depth interviews) and quantitative approaches (e.g. psychometric testing) to develop and standardize measures for use by health researchers especially from LMICs. In the industrialized setting, we recommend the use of YRBSS or HBSC owing to their comprehensive approach to assessing multiple forms of HRB. The results of more research on HRB among chronically ill adolescents could translate to significant clinical, public health and social economic benefits, especially for adolescents living with such illnesses and their families.

## Abbreviations

ACASI: Audio Computer Assisted Self Interview
AUDIT: Alcohol Use Disorder Identification Test
CAPI: Computer Assisted Personal Interview
CRAFT: car, relax, alone, forget, friends, trouble
HBSC: Health Behavior in School-aged Children
HIV: human immunodeficiency virus
HRB: health risk behavior
LMIC: low and middle income countries
SMASH: Swiss Multi-centric Adolescent Survey on Health
YRBSS: Youth Risk Behavior Surveillance System
